# Generation and characterization of a Tet-On (rtTA-M2) transgenic rat

**DOI:** 10.1186/1471-213X-10-17

**Published:** 2010-02-16

**Authors:** Yi Sheng, Chih-Cheng Lin, Junming Yue, Meena Sukhwani, Jennifer J Shuttleworth, Tianjiao Chu, Kyle E Orwig

**Affiliations:** 1 Department of Obstetrics, Gynecology and Reproductive Sciences, University of Pittsburgh School of Medicine, Pittsburgh, PA 15213, USA; 2 Magee-Womens Research Institute, Pittsburgh, PA 15213, USA; 3 Current address: Department of Physiology, University of Tennessee, Memphis, TN 38163, USA

## Abstract

**Background:**

The tetracycline-inducible gene regulation system is a powerful tool that allows temporal and dose-dependent regulation of target transgene expression in vitro and in vivo. Several tetracycline-inducible transgenic mouse models have been described with ubiquitous or tissue-specific expression of tetracycline-transactivator (tTA), reverse tetracycline-transactivator (rtTA) or Tet repressor (TetR). Here we describe a Tet-On transgenic rat that ubiquitously expresses rtTA-M2 driven by the murine ROSA 26 promoter.

**Results:**

The homozygous rat line (ROSA-rtTA-M2) generated by lentiviral vector injection, has a single integration site and was derived from the offspring of a genetic mosaic founder with multiple transgene integrations. The rtTA-M2 transgene integrated into an intron of a putative gene on chromosome 2 and does not appear to affect the tissue-specificity or expression of that gene. Fibroblasts from the ROSA-rtTA-M2 rats were transduced with a TetO_7_/CMV-EGFP lentivirus and exhibited doxycycline dose-dependent expression of the EGFP reporter transgene, in vitro. In addition, doxycycline-inducible EGFP expression was observed, in vivo, when the TetO_7_/CMV-EGFP lentivirus was injected into testis, kidney and muscle tissues of ROSA-rtTA-M2 rats.

**Conclusions:**

This conditional expression rat model may have application for transgenic overexpression or knockdown studies of gene function in development, disease and gene therapy.

## Background

Rats are widely used models of basic biology, human physiology and disease, but the study of genes and their functions in rats lags far behind progress in mice. During the 1980s, mice became the predominant model for functional genetic studies because progress culturing and manipulating eggs and embryos [1] of this species facilitated pronuclear injection and blastocyst injection, ex vivo [2-8]. In addition, development of germline competent embryonic stem cells [9,10] enabled complex and targeted genetic manipulations (e.g., knockin and knockout). In contrast, pronuclear injection is generally considered less efficient in rats [11], germline competent ES cells were only recently established for this species [12,13] and there are no reports of gene targeting in ES cells to produce knockout rats.

Rats are particularly amenable to laboratory investigations because of their intermediate size, short gestation and high fecundity. Rats are about ten times larger than mice and thus, provide more tissue and are more amenable to surgical manipulation, vascular access and repeated sampling. Due in part to these features, the rat is a valuable model for transplantation, cardiovascular, hypertension, neuroscience, pharmacology, diabetes, obesity, aging and cancer [14,15]. Furthermore, there is over a century of phenotypic and physiological data on anatomy, physiology and disease for various inbred rat models [15,16]. Combining these data with improved genetic and genomic resources will provide unprecedented opportunities for functional genetic studies in this species. Recognizing this opportunity, NIH has funded the Rat Expressed Sequence Tag (EST) Program, the Rat Genome Program and the Rat Genome Database and established the Rat Resource Research Center (RRRC; http://www.nrrrc.missouri.edu) during the past decade [17]. This NIH effort is matched by the National Bio Resource Project for the Rat (NBRP-Rat; http://www.anim.med.kyoto-u.ac.jp/NBR/) in Japan and rat resource repositories in Europe (http://www.mh-hannover.de/2652.html and http://www.euratools.eu). Thus, there is a strong impetus to expand the transgenic tools for this species.

Production of transgenic rats by pronuclear injection [18,19] is now routine, albeit less efficient than for the most efficient mouse strains [14]. In addition, lentiviruses [20,21], sperm [22] and spermatogonial stem cells [23,24] have recently been exploited as vehicles for modifying the rat germline and producing transgenic rats with efficiencies ranging from 6-46%. Although germline competent ES cells have not yet been used to produce knockout rats, transgenic expression of short hairpin RNAs (shRNA) or microRNAs (miRNA) allow knockdown of target gene expression for investigations of gene function [25,26]. Chemical mutagenesis of germ cells by N-ethyl-n-nitrosourea (ENU) can introduce random point mutations in rat genome, but the requirement of labor-intensive screening analysis and high cost limits its application [27,28]. Finally, pronuclear injection of zinc-finger nucleases is a new tool to generate knockout rats [29]. Thus, several approaches are now available to modify and manipulate the rat genome/transcriptome and expand the transgenic resources for this species.

Some transgenic manipulations have developmental effects (e.g., embryonic lethality), which preclude studies in adult tissues. These problems can be circumvented through the use of tissue-specific and/or conditional promoters. Several inducible promoter systems have been described, including those regulated by tetracycline [30], ecdysone [31], rapamycin [32], or steroids [33]. The tetracycline (Tet)-inducible system has been the most extensively characterized and allows conditional regulation of gene expression in vitro [30] and in vivo [34,35]. Tet-repressor (TetR), Tet transactivator (tTA) and reverse Tet transactivator (rtTA) systems mediate tetracycline (or doxycycline)-controlled transactivation of a second recombinant transgene located downstream of composite promoters that contain tet operator (TetO) sequences (e.g., TetO_7_/CMV). To date, over 100 different tetracycline-regulated transgenic mouse lines have been described (reviewed at the Tetmouse Base, http://www.zmg.uni-mainz.de/tetmouse/index.htm). In contrast, only a few rat lines have been generated, expressing either tTA [36-38] or rtTA [39]. To facilitate loss-of-function studies, two conditional (inducible) transgenic rats have been described in which TetR/TetO system is employed to regulate expression of an shRNA [40,41].

Here we describe the generation and characterization of a novel Tet-On transgenic rat. This transgenic line was generated by injecting lentiviral vectors containing the ubiquitous ROSA26 promoter [42] and an rtTA-M2 transgene under the zona pellucida of one-cell rat embryos, as previously described [20]. rtTA-M2 is a mutant of rtTA that has increased stability, reduced background expression in the absence of doxycycline (Dox, tetracycline analog) and improved inducibility in the presence of Dox [43]. ROSA-rtTA-M2 rats generated in this study exhibit wide-spread expression of rtTA-M2 and Dox-regulated transactivation of a target TetO_7_/CMV-EGFP transgene. The TetO_7_/CMV composite response element is commonly referred to as pTet or TRE.

## Results

**Figure 1:**
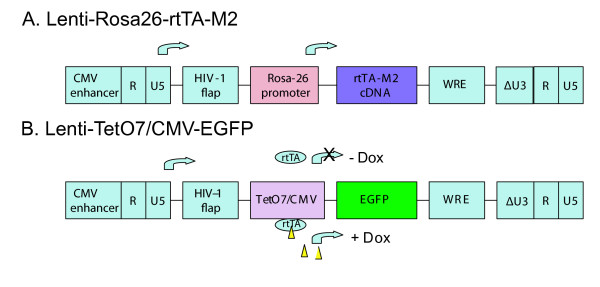
**pLenti-Rosa26-rtTA-M2 and pLenti-TetO_7_/CMV-EGFP lentiviral vectors**. Replication-deficient lentiviral vectors feature a self-inactivating 3'UTR and a CMV enhancer replaces the U3 region of the 5'UTR. WRE: woodchuck hepatitis posttranscriptional regulatory element. **A**. Expression of rtTA-M2 is driven by the ubiquitous Rosa-26 promoter. **B**. Expression of EGFP is under control of a seven repeat Tet operator and CMV minimal promoter (TetO_7_/CMV). In the presence of doxycycline (yellow triangles), The rtTA-M2 protein (blue ovals) encoded by the construct in (A) binds the TetO_7 _operator and transactivates EGFP expression from the construct in (B).

**Figure 2:**
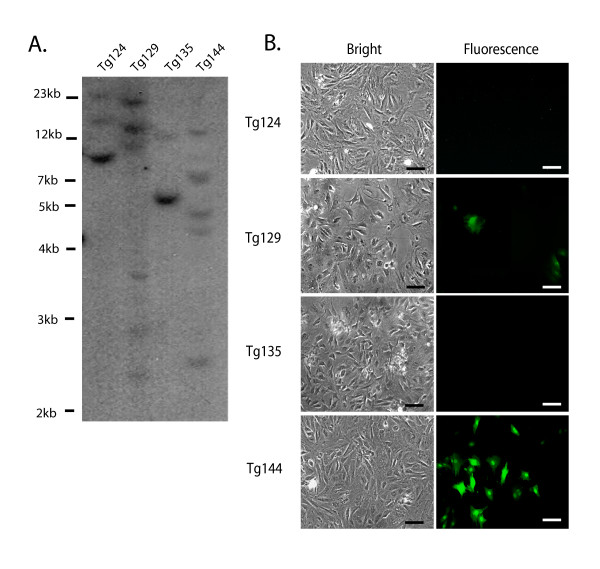
**Integration site(s) and transgene expression analysis of rtTA-M2 transgenic rat founders**. **A**. Southern blot analysis. Genomic DNA from four transgenic founder animals (Tg124, Tg129, Tg135, Tg144) was digested with EcoRI and hybridized with rtTA-M2 cDNA. **B**. Expression and function analysis of the rtTA-M2 transgene. Tail fibroblasts from the four rtTA-M2 transgenic founders were transduced with Lenti-TetO_7_/CMV-EGFP and cultured in the presence of Dox (500 ng/ml). EGFP expression was observed by immunocytochemistry 48 hours post-transduction using an epifluorescent microscope and a FITC filter cube. EGFP expression was observed in transgenic founders, Tg129 and Tg144, indicating expression of the rtTA-M2 transgene and transactivation of the TetO_7_/CMV-EGFP transgene. EGFP expression was not detectable in tail fibroblasts from transgenic founders Tg124 and Tg135. EGFP expression was not observed in fibroblasts from any of the transgenic founders if Dox was not added. Bar = 20 μm.

The ROSA26-rtTA-M2 lentiviral vector (Figure 1A) was injected under the zona pellucida of 219 1-cell S/D rat embryos. A total of 179 injected embryos (81.7%) cleaved to produce 2-cell embryos after overnight culture and these were transferred to the oviducts of 3 pseudopregnant S/D female rats. These transferred embryos resulted in 35 live progeny and RT-PCR results (not shown) indicated that 12 (34%) of these carried the ROSA26-rtTA-M2 transgene. Four founder progeny (Tg124, Tg129, Tg135, Tg144) were maintained for further characterization. Southern blot analyses indicated that founder animals had between two (Tg135) and six (Tg129) proviral insertion sites (Figure 2A). The intensity of bands corresponding to independent insertions were varying in some cases, indicating they could be genetic mosaics.

Cultures of tail fibroblasts were established for each founder animal and were transduced with a lentivirus containing the TetO_7_/CMV-EGFP reporter transgene (Figure 1B) in the presence of Dox. EGFP fluorescence was observed 48 hours later in fibroblasts from two founders (Tg129 and Tg144) using an epifluorescent microscope (Figure 2B). EGFP expression was not observed in the absence of Dox (not shown). The founder Tg144, with five proviral integrations and the most robust Dox-dependent EGFP expression, was selected to establish a ROSA26-rtTA-M2 transgenic rat line. This founder was bred to wild type S/D females to produce F1 transgenic progeny and segregate the independent integration sites.

**Figure 3:**
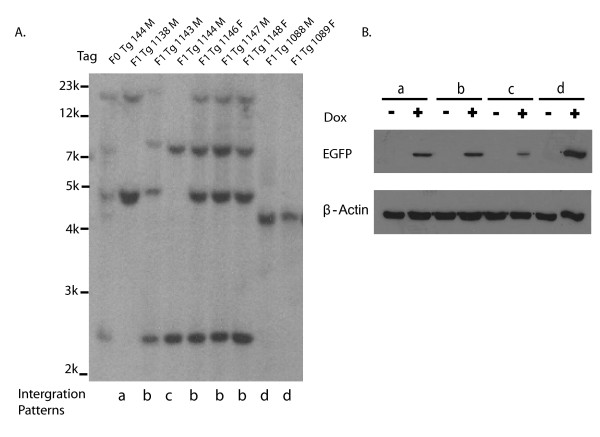
**DNA and transgene expression analysis of F1 progeny from transgenic founder Tg144**. **A**. Southern blot analysis. Samples of genomic DNA from Tg144 and its F1 progeny were digested with EcoRI and hybridized with rtTA-M2 cDNA. Founder (F0) and first generation progeny (F1) identification numbers are indicated at the top of each lane. Four different transgene integration patterns were passed to the F1 progeny of transgenic founder Tg144 and are identified by letter at the bottom of each lane (a, b, c, d). F1 progeny carried one to four integrations of the rtTA-M2 transgene. **B**. Fibroblasts from F1 progeny with each of the four integration patterns were transduced with Lenti-TetO_7_/CMV-EGFP and cultured with or without Dox (Dox +/-). EGFP expression was evaluated by Western blot analysis 48 hours after viral transduction. The blot was stripped and reprobed with β-actin antibody as a loading control.

**Figure 4:**
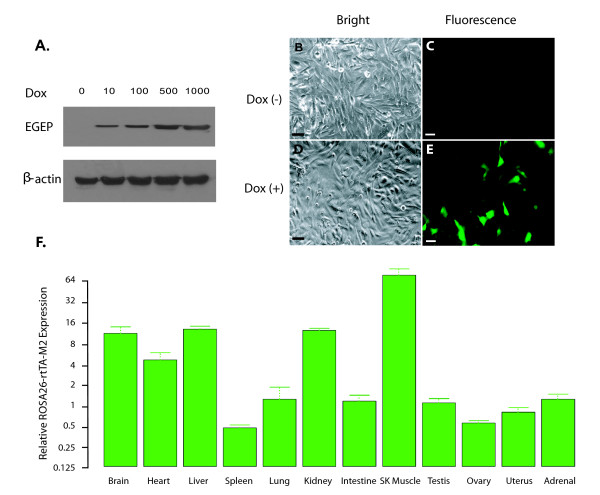
**Characterizing rtTA-M2 transgene expression from homozygous ROSA26- rtTA-M2-1089/1088 transgenic rats (single integration)**. A homozygous rtTA-M2 transgenic rat line (ROSA26-rtTA-M2-1089/1099; renamed ROSA-rtTA-M2) was established by breeding F1 Tg1088 (male) and F1 Tg1089 (female), which each exhibited a single common integration of the ROSA26-rtTA-M2 transgene. **A**. Doxycycline dose response analysis. Fibroblasts from ROSA-rtTA-M2 F2 progeny were transduced with Lenti-TetO_7_/CMV-EGFP and cultured in medium containing different concentrations of Dox (0 to 1000 ng/ml). Western blot analyses were used to evaluate EGFP expression. The blot was stripped and reprobed with β-actin antibody as a loading control. **B-E**. rtTA-M2 gene was stably transmitted. Fibroblasts from the F5 generation of homozygous ROSA-rtTA-M2 rats were transduced with Lenti-TetO_7_/CMV-EGFP and cultured in medium without (B and C) or with (D and E) Dox (500 ng/ml). EGFP expression was observed by immunocytochemistry 48 hours post-transduction (Bar = 20 μm). **F**. Real time-PCR analysis to determine tissue distribution of rtTA-M2 expression from ROSA-rtTA-M2 rats. rtTA-M2 expression in each tissue is presented relative to the rtTA-M2 expression in the testis. The whiskers represent the standard errors. β-actin was used as an internal control for each tissue.

All transgene insertions from founder (F0) 144 were transmitted through the germline and four different integration patterns were recovered in the F1 progeny (Figure 3A). Cultures of tail fibroblasts established for each of the F1 progeny were transduced with Lenti-TetO_7_/CMV-EGFP and maintained in the presence or absence of Dox for 48 hours. Western blot analyses demonstrated Dox-dependent expression of the EGFP reporter gene from progeny with each integration pattern and revealed that expression level was not dependent on the number of integration sites (Figure 3B). Indeed, F1 progeny with only a single integration (integration pattern D) exhibited the highest level of Dox-dependent EGFP expression (Figure 3B). F1 female 1089 and F1 male 1088 had a common single integration site corresponding to integration pattern D and were bred together to establish the homozygous transgenic line ROSA-rtTA-M2-1089/1088 (ROSA-rtTA-M2). Cultures of tail fibroblasts established from homozygous F2 progeny exhibited tight regulation and Dox dose-dependent induction of the TetO_7_/CMV-EGFP reporter transgene (Figure 4A). Immunocytochemical analysis of GFP expression in cultures of tail fibroblasts from the F2-F5 generation of homozygous ROSA-rtTA-M2 rats demonstrated that the transgene is stably integrated, transmitted through the germline and not silenced (results from the F5 generation are depicted in Figure 4B-E). Rats from this line exhibited normal fertility and appeared phenotypically normal. Real-time PCR analysis was used to quantitatively evaluate rtTA-M2 expression in multiple tissues. From the twelve tissues analyzed, the highest rtTA-M2 expression was observed in skeletal muscle, followed by liver, kidney, brain, heart, adrenal, lung, intestine, testis, uterus, ovary and spleen (Figure 4F).

**Figure 5:**
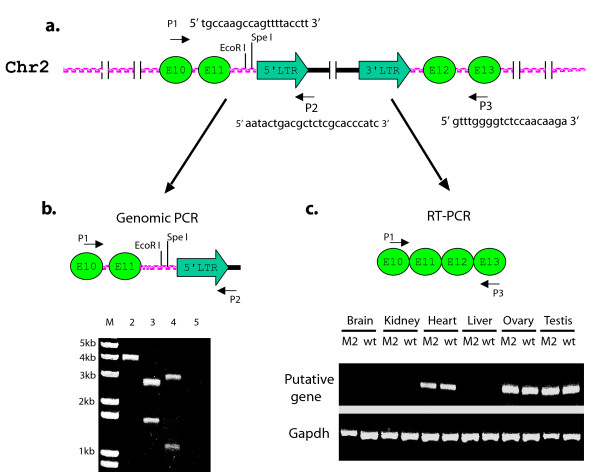
**Rosa-rtTA-M2 integrated into a putative gene on chromosome 2**. **A**. Rosa-rtTA-M2 provirus inserted into intron 11 of putative gene (GeneID: 310880) on chromosome 2. E: Exon. **B**. Genomic PCR using primers from Exon 10 (P1) and vector (P2) verified this integration. Lane M: 1 Kb DNA ladder; lane 2, no digestion; lane 3, EcoRI digestion; lane 4, Spe I digestion; lane 5, wide type rat DNA). **C**. Detection of the putative endogenous gene expression in rat tissues by RT-PCR. Reverse transcription was performed with an oligo (dT)_17 _primer. The endogenous transcript was amplified using primers from exon 10 (P1) and exon 13 (P3) of this putative gene. M2: ROSA-rtTA-M2 rat; wt: wild type rat.

Linker-mediated PCR was used to amplify the junctions between chromosomal DNA and the ROSA26-rtTA-M2 transgene (see Additional File 1: Figure S1 for summary of Linker-mediated PCR). Sequence comparison with the NCBI rat genomic database indicated that the transgene integrated into intron 11 of a putative gene (GeneID: 310880) on chromosome 2 (Figure 5A). The integration site was further confirmed by PCR amplification of the junctions between genomic and transgene DNA (Figure 5B). Amplification of the 5' junction produced a 4 kb product (Figure 5B, lane 2), which was cleaved to the expected smaller products by EcoR1 (Figure 5B, lane 3) and Spe1 (Figure 5B, lane 4). RT-PCR was performed to compare expression of the putative gene (GeneID: 310880) in tissues of wild type and ROSA-rtTA-M2 rats. Expression of the putative gene was observed in heart, ovary and testis, but not brain, kidney or liver. There were no differences in expression of the putative gene between wild type and transgenic rats (Figure 5C).

**Figure 6:**
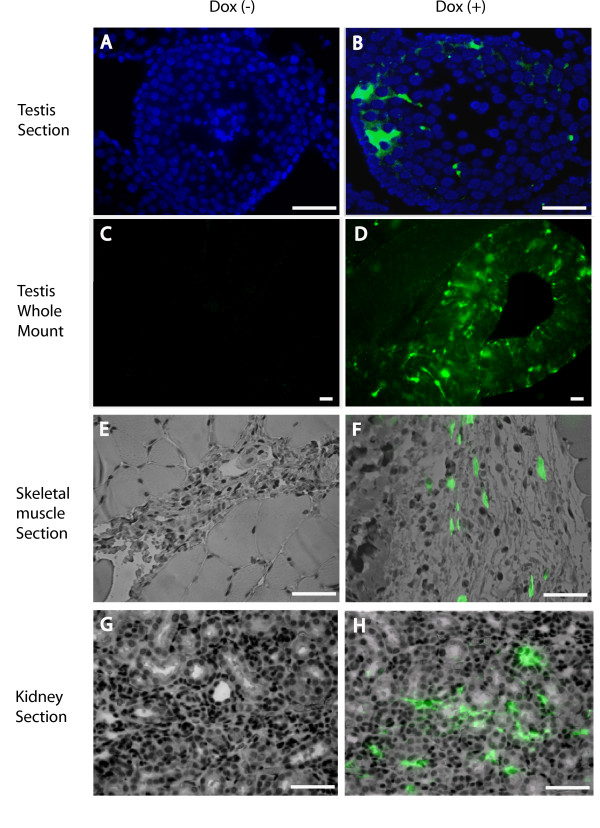
**Evaluation of EGFP expression in tissues from ROSA-rtTA-M2 transgenic rat**. Lenti-TetO_7_/CMV-EGFP was injected into the testis, hind leg skeletal muscle or kidney of ROSA-rtTA-M2 rats without (A, C, E and G) or with (B, D, F and H) Dox in their drinking water (0.5 mg/ml). **A-B**. Immunohistochemical analysis of EGFP expression in histological section of testicular seminiferous tubule. Counterstain: DAPI. **C-D**. Immunohistochemical analysis of EGFP expression in whole mount of testicular seminiferous tubule. **E-F**. Immunohistochemical analysis of EGFP expression in skeletal muscle. **G-H**. Immunohistochemical analysis of EGFP expression in Kidney. Counterstain: hematoxylin. Bar = 50 μm (all panels).

To test the function of the rtTA-M2 transgene in vivo, the TetO_7_/CMV-EGFP reporter lentivirus was injected into testes, skeletal muscle (hind leg) and kidney of ROSA-rtTA-M2 rats maintained with or without Dox in the drinking water. Based on real time PCR results in Figure 4F, these represent low (testis), medium (kidney) and high (skeletal muscle) expressing tissues. Dox-dependent expression of EGFP was observed in testis (Figure 6A-D), skeletal muscle (Figure 6E-F) and Kidney (Figure 6G-H). EGFP expression was not observed for any of these tissues in the absence of Dox (Figure 6A, C, E and 6G).

## Discussion

In this study, we generated a "Tet-On" transgenic rat line that exhibits ubiquitous expression of the modified reverse tetracycline-dependent transactivator, rtTA-M2, under control of the ROSA26 promoter. As detailed in the introduction, it is the dawn of a new era for biological investigations using the rat model. Vast phenotypic and physiological data generated over the past century on rat anatomy, physiology and disease can now be combined with new genetic resources to enable state of the art functional genetic investigations. The best way to study gene function is through the generation of gain-of-function (transgenic) or loss-of-function (knock-out, Knock-down) animal models. The functions of over 5,000 genes have been investigated using transgenic or knockout approaches in the mouse (Mouse Genome Database, [44]). Considerably fewer genes in the rat genome have been studied functionally using gain-of-function or loss-of-function mutants. This deficit in rats may arise in part from the fact that traditional transgenic approaches in rat are less efficient than in mouse and in part from the fact that germline competent rat embryonic stem (ES) cell lines were not available, until recently [12,13]. To date, there are no reports that these rat ES cells have been used for targeted inactivation of specific genes and generation of knockout rats. However, Geurts and co-workers recently reported the generation of targeted mutant rats by injection of zinc finger nucleases in embryos. This may constitute a powerful approach for generating loss-of-function rat models and has the potential for widespread application if it can be combined with homologous recombination [29,45].

Several approaches have been used to generate transgenic rats, including pronuclear injection, ICSI [22,46], spermatogonial stem cells [23,24] and lentiviral injection into one cell embryos [20,21]. These approaches have varying efficiencies, strengths and weaknesses. No single approach is superior for every application. In the current study, Tet-On transgenic rats were generated by injecting lentiviral vectors (carrying the ROSA-rtTA-M2 transgene) under the zona pellucida of one cell embryos. This approach was initially described for rats by Lois and co-workers [1] and is significantly more efficient (34% for the current study) than the standard pronuclear injection method. Lentivirus-mediated transgenesis does have limitations. The transgene cassette inserted between the viral LTRs is typically limited to less than 10 Kb. Viral genomes with larger transgenes are not packaged efficiently, leading to decreased viral titer. The ROSA-rtTA-M2 transgene cassette was approximately 1.5 Kb, well within the capacity of the lentiviral genome.

A second limitation of lentivirus-mediated transgenesis is that each lentiviral integration contains a single copy of the transgene, rather than head to tail arrays of multiple transgenes that can occur after pronuclear injection. This may be limiting if high levels of transgene expression are required. Also, multiple integrations in a founder generated by lentiviral injection will be segregated in the next generation. For example, founder Tg144 with five transgene integrations produced progeny with four different integration patterns (Figure 3A).

Integration site effects were also revealed in the F1 progeny from founder Tg144. F1 progeny with integration pattern d (single integration) exhibited stronger expression of the rtTA-M2 transgene than F1 progeny with integration pattern b (four integrations; Figure 3B). Expression of rtTA-M2 from the single homozygous integration in the ROSA-rtTA-M2 line was sufficient to drive expression from the Lenti-TetO_7_/CMV-EGFP reporter gene, in vitro (Figure 4A-E) and in vivo (Figure 6).

Functional genetic studies can be complicated if the genetic modification causes an embryonic lethal phenotype. This complication can be circumvented by using conditional expression systems that allow the target transgene to be activated at specific times of development. In the current study we utilized a mutant of the reverse Tet transactivator (rtTA-M2; [43]) that activates gene expression by binding to the Tet operator (TetO) in the presence of doxycycline (Tet-On). The M2 iteration of rtTA is stable in mammalian cells (HeLa), functions at a 10-fold lower concentration of Dox than rtTA and causes no background expression [43]. We found that rtTA-M2 exhibited similar characteristics in rat primary fibroblasts and in vivo using the ROSA-rtTA-M2 line. This transgenic rat line has been bred to homozygosity and has been propogated to the fifth generation without silencing of transgene expression. Early this year, three groups have reported generating transgenic rats by driving TetR expression with the chicken β-actin or ubiquitin promoters [38,41] or tTA by the chicken β-actin promoter [40]. In addition, Konopka and coworkers [39] generated a "Tet-On" transgenic rat in which rtTA is driven by the EF1α promoter. The EF1α promoter is typically expected to cause ubiquitous expression, but functional rtTA expression (indicated by activation of a TetO/CMV-EGFP reporter) was limited to the testis in that study.

## Conclusion

The ROSA-rtTA-M2 Tet-On rat line may provide a versatile tool for functional genetic studies because rtTA is ubiquitously expressed and can regulate conditional expression of target transgenes, in vitro or in vivo. In the current proof-of-principle study, an EGFP reporter gene was placed downstream of Tet operator sequences and CMV minimal promoter (TetO_7_/CMV) in a lentiviral vector. Similar constructs can be used for conditional over-expression or knock-down (using shRNA or miRNA sequences [40]) by replacing EGFP with any transgene of interest. Functions of target genes can be evaluated in vivo by injecting the target transgene into tissues of ROSA-rtTA-M2 rats as demonstrated here or by breeding ROSA-rtTA-M2 rats to a second transgenic line containing the TetO_7_/CMV minimal promoter and a transgene of interest. The utility and flexibility of the Tet regulatory system in rats will be increased by continuing to generate various iterations of Tet repressors, Tet transactivators and Tet responsive promoters, including the use of alternative ubiquitous or tissue-specific promoters.

## Methods

### Lentiviral construct and virus production

The Dox-regulated vector system consists of two lentiviral vectors, Lenti-ROSA26-rtTA-M2 and Lenti-TetO_7_/CMV-EGFP (Figure 1). Both vectors were constructed using the pC-FUW lentiviral backbone [20]. Lenti-ROSA26-rtTA-M2 was generated by isolating the ROSA26-rtTA-M2 sequence from PBII/Rosa26/M2/HGHpA plasmid (gift from Dr. Richard Chailet, University of Pittsburgh) and ligating this sequence into the PacI/EcoRI sites of pC-FUW, replacing the original UBC-EGFP sequence. Lenti-TetO_7_/CMV-EGFP was generated by isolating the TetO_7_/CMV sequence from pTight-TRE (Clontech, Mountain View, CA) and inserting this sequence into PacI/BamH1 sites of pC-FUW. The reporter EGFP gene fragment was inserted into BamH1/EcoR1 sites, which is under control of TetO_7_/CMV promoter.

The VSV-G peudotyped lentiviral particles were generated by using 293FT cells and the ViraPower Lentiviral Expression System (Invitrogen, Carlsbad, California). Lentivirus supernate was collected 72 hours after transfection, passed through a 0.4 μm filter unit and concentrated by ultracentrifugation at 50,000 g, 4°C for 2.5 hours. Pellets were resuspended in 200 ul (1/150 of the original volume) culture medium or PBS. Lentivirus titers were determined by p24 ELISA (Cell Biolabs, Inc, San Diego, CA).

### Generation of ROSA26-rtTA-M2 transgenic rats

Female Sprague-Dawley rats (S/D, 4-5 week old) were superovulated by injection with pregnant mare serum gonadotropin (PMSG, 20 IU, IP), followed 48 hours later by injection with human chorionic gonadotropin (hCG, 20 IU, IP). Immediately after hCG injection, superovulated females were housed with fertile S/D males overnight. Females with vaginal plugs were euthanized the next morning for collection of one-cell embryos. Lentiviral supernate (~10 pl) was injected into the perivitelline space of the one-cell embryos and the embryos were cultured overnight in KSOM medium (Minipore, Billerica, MA) supplemented with essential amino acids (Invitrogen). Embryos that developed to 2-cell stage were transferred into the oviducts of peudopregnant recipient rats, as previously described [47]. All animal procedures were approved by the Institutional Animal Care and Use Committee (IACUC) of the University of Pittsburgh, which is the IACUC of record for Magee-Womens Research Institute (Assurance number A3654-01), in accordance with the National Institutes of Health Guidelines for the Care and Use of Laboratory Animals.

### Screening for identification of founder transgenic rats

To identify founder (F0) transgenic rats, tail biopsies were collected at two weeks of age for genomic DNA isolation using Gentra Puregene Mouse Tail Kit (Qiagen). The resulting genomic DNA samples were screened by polymerase chain reaction (PCR) and Southern blot analysis for the rtTA-M2 transgene. The primers for PCR were: forward, 5'TTACCCGGGGAGCATGTCAAGG3' and reverse, 5'CCACCATGTCTAGACTGGACAAGAGC3'. For Southern blot analysis, 15 μg of each DNA sample was digested with EcoR1, electrophoresed on 0.8% agarose gel and transferred to a Nytran^@^Supercharge membrane (Whatman Inc, Sanford, ME). The membranes were then hybridized overnight at 67°C with Rapid-hyb buffer (Amersham, Piscataway, NJ) containing labeled rtTA-M2 cDNA (RadPrime DNA Labeling System kit, Invitrogen).

A portion of each rat tail biopsy was minced and digested (37°C, 15 min) in 3 ml of 0.25% trypsin (Invitrogen) to generate a suspension of tail fibroblasts. The digestion was terminated by addition of DMEM containing 10% FBS and the cell suspension was centrifuged (600 g, 5 min). The resulting cell pellets were resuspended in 2 ml DMEM culture medium with 10% FBS (Hyclone, Logan, Utah). Undigested tissues were allowed to sediment to the bottom of the tube for two minutes and supernates were transferred to a 12-well plate to establish cultures of tail fibroblasts.

The fibroblasts from the rat tails which carried the rtTA-M2 transgene (determined by PCR genotyping) were passaged and transduced with Lenti-TetO_7_/CMV-EGFP in the presence of 6 μg/ml polybrene (Sigma-Aldrich, St. Louis, MO). Following transduction, fibroblasts were maintained in medium containing doxycycline (Dox, 500 ng/ml, Sigma-Aldrich). Control cultures for each founder were maintained in medium without Dox. GFP expression was evaluated 72 hours after transduction using an epifluorescent microscope and FITC filter cube. Founders with the highest Dox-dependent GFP expression were bred with wild type S/D rats and the offspring were further evaluated by Southern blot and fibroblast culture to characterize and segregate individual transgene integrations.

### Determination of Lenti-ROSA26-rtTA-M2 integration site

The integration site of the ROSA26-rtTA-M2 transgene was determined by linker-mediated (LM)-PCR [48,49]. Genomic DNA samples (2 μg) were digested with NlaIII (20 u) for 1 hour followed by purification using a PCR clean-up kit (Qiagen, Valencia, CA). The products were then ligated on each end with a linker containing T7 and Sp6 promoter sequences (see Additional File 1: Figure S1, step 2) followed by purification using the PCR clean-up kit (Qiagen). Primer extension was performed using the lentiviral vector specific primers, followed by two rounds of PCR using vector specific primers and primers from the linker (complementary to the T7 and Sp6 promoter sequences). The final PCR products were gel purified and subcloned into pCR^® ^II-TOPO^® ^vector (Invitrogen) for sequencing. The integration site was determined by analyzing the sequence with the NCBI Rat Genomic Sequence Blast tool (http://www.ncbi.nlm.nih.gov/genome/seq/BlastGen/BlastGen.cgi?taxid=10116, [50]). The details of these steps and oligonucleotide sequences were shown in Additional File 1: Figure S1.

### Detection of rtTA-M2 expression in tissues

Real Time-polymerase chain reaction (RT-PCR) was used to detect rtTA-M2 expression in different tissues. Total RNA from each tissue was extracted by Trizol^® ^Reagent (Invitrogen). Reverse transcription (RT) was performed using SuperScript III reverse Transcriptase (Invitrogen) with Oligo (dT)_17_. For SYBR Green real-time PCR, 1 μl of RT product was diluted three times and used as a template in each reaction. PCR reactions were performed in triplicate for each tissue sample. Independent tissue samples were obtained from three ROSA-rtTA-M2 rats. Primers for rtTA-M2 were forward, 5'AGGCTGGACAAGAGCAAAGT and reverse, 5'ACAGGGTAGGCTGCTCAACT3'. β-actin was amplified as an internal control using the primers: forward, 5'AGGAGCGTAAAAGTTTCTCCAAG and reverse, 5'CCAGGGATAACTGCAAGGGC3'. The SYBR Green-based real-time PCR was performed using the LightCycler 4800 real-time PCR system (Roche Applied Science; Indianapolis, IN). The PCR conditions were as follows: denature 10 sec at 95°C, anneal 30 sec at 60°C, extend 10 sec at 72°C, repeat 35 cycles. The relative expression of rtTA-M2 in each tissue was calculated using the ΔΔCt method where the mean Ct of rtTA-M2 for each sample was divided by the mean Ct of β-actin for that sample. The resulting ΔCt values were used to calculate rtTA-M2 expression in each tissue relative to rtTA-M2 expressing in the testis using the formula: 2^-ΔCt Transgenic Tissue-ΔCt Transgenic testis^.

### Western Blot analysis

Fibroblasts were lysed in lysis buffer (50 mM Tris-HCl, pH 7.5, 150 mM NaCl, 0.1% SDS, 1% NP-40, 0.5% deoxycholate), protein (20 μg) from each sample was separated on a 10% denaturing polyacrylamide gel and transferred to nitrocellulose membranes (Whatman Inc.). The blots were probed with GFP antibody (Clontech), and reprobed with β-actin antibody (Sigma-Aldrich). GFP immunoreactivity was detected using the SuperSignal West Pico Chemiluminescent Substrate (Piece, Rockford, IL)

### In vivo analysis and immunohistochemistry

The transgenic rats were anaesthetized and Lenti-TetO_7_/CMV-GFP was injected into skeletal muscle (hind legs), kidney and testis seminiferous tubules by efferent duct injection, as previously described [51]. Treated animals were given water containing 0.5 mg/ml Dox and 2.5% sucrose for 10 days (ad libetum). The control group was not treated with Dox. Following treatment, testis tissue was prepared by removing the tunica albuginea and treating briefly with collagenase IV (1 mg/ml, Sigma) to disperse seminiferous tubules. Following treatment, GFP expression in whole mount preparations of muscle, kidney and testis tissues was observed directly, using an epifluorescent microscope and a FITC/TRITC dual emission filter cube. The dual emission filter cube is used to distinguish GFP fluorescence (green) from autofluorescence (red, [52]). Muscle, kidney and testis tissues were also fixed with 4% paraformaldehyde and prepared for immunohistochemical analysis of GFP expression in section. Tissue sections (5 μm) were deparaffined, rehydrated and subjected to antigen retrieval in Sodium Citrate Buffer (10 mM Sodium Citrate, 0.05% Tween, pH6.0) at 97°C, 30 minutes. Sections were hybridized with a mouse anti-EGFP monoclonal antibody (1:300 dilution, Clontech) for 90 min at room temperature. Normal mouse IgG was used in place of the anti-EGFP primary antibody for control sections. Sections were washed (3×) with PBS containing 0.1% Tween-20 (PBST), followed by incubation with an Alexa Fluor^® ^488-conjugated goat anti-mouse secondary antibody (Invitrogen) for 60 min. Sections were washed and mounted under glass cover slips with Vectashield mounting medium containing DAPI (Vector Laboratories Inc., Burlingame, CA) or Hematoxylin (Sigma).

## Authors' contributions

YS designed experiments, analyzed data and wrote the draft. CCL performed embryo manipulations and helped with genotyping. MS performed efferent duct injections. JY generated the lentivirus and performed real-time PCR. JJS provided animal husbandry and helped with genotyping. TJC performed statistical analysis. KEO supervised this project, helped design experiments, analyzed data and edited the final manuscript. All authors have read and approved the final manuscript.

## Supplementary Material

Additional file 1 **Figure S1. Diagram of cloning Lenti-Rosa26-rtTA-M2 integration site**. Genomic DNA is partially digested with Nla III, then ligated to a linker containing T7 and Sp6 promoter sequences. Provirus integration site sequence is amplified by primer extension from vector sequence and followed by two round of PCR with vector specific, T7 and Sp6 primers. PCR products are gel purified, subcloned into pCR^®^2.1-TOPO^® ^and sequenced. A BLAST search of the rat genome was performed to identify homologous sequence. Click here for file
