# Supplementary material for: Generation and characterization of a Tet-On (rtTA-M2) transgenic rat
Source: BMC Dev Biol. 2010 Feb 16;10:17. doi: 10.1186/1471-213X-10-17 (PMC2834583; doi:10.1186/1471-213X-10-17)
Supplement: Additional file 1 — Figure S1. Diagram of cloning Lenti-Rosa26-rtTA-M2 integration site. Genomic DNA is partially digested with Nla III, then ligated to a linker containing T7 and Sp6 promoter sequences. Provirus integration site sequence is amplified by primer extension from vector sequence and followed by two round of PCR with vector specific, T7 and Sp6 primers. PCR products are gel purified, subcloned into pCR®2.1-TOPO® and sequenced. A BLAST search of the rat genome was performed to identify homologous sequence. [file 1471-213X-10-17-S1.PDF]

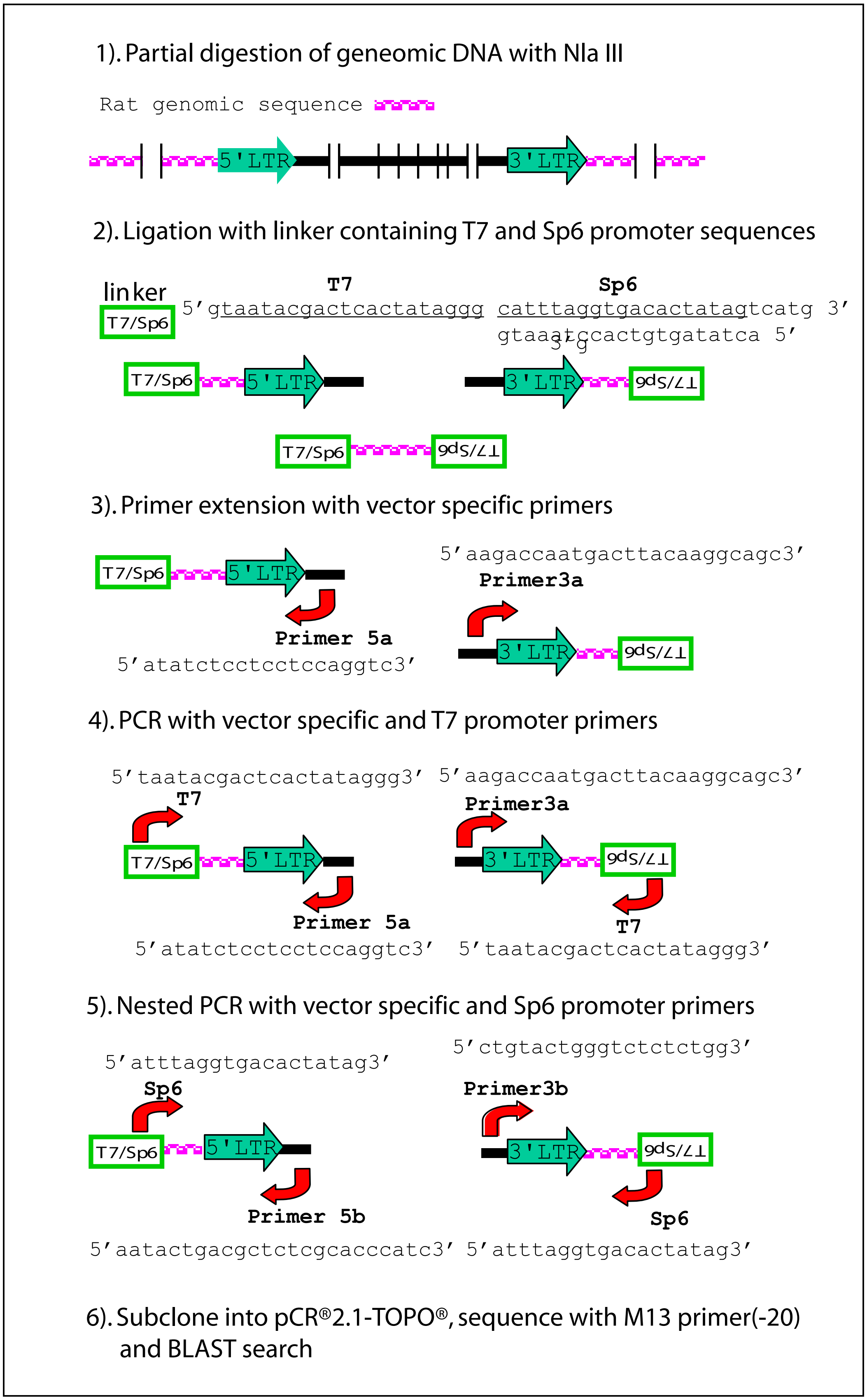

1). Partial digestion of geneomic DNA with Nla III
Rat genomic sequence
5'LTR
3'LTR
2). Ligation with linker containing T7 and Sp6 promoter sequences
linker
T7/Sp6
T7
Sp6
5'gtaatacgactcactataggg catttaggtgacactatagtcatg 3'
3'g
gtaaatccactgtgatatca 5'
T7/Sp6
5'LTR
3'LTR
T7/Sp6
T7/Sp6
T7/Sp6
3). Primer extension with vector specific primers
T7/Sp6
5'LTR
Primer 5a
5'atatctcctcctccaggtc3'
5'aagaccaatgacttacaaggcagc3'
Primer3a
3'LTR
T7/Sp6
4). PCR with vector specific and T7 promoter primers
5'taatacgactcactataggg3'
T7
T7/Sp6
5'LTR
Primer 5a
5'atatctcctcctccaggtc3'
5'aagaccaatgacttacaaggcagc3'
Primer3a
3'LTR
T7/Sp6
T7
5'taatacgactcactataggg3'
5). Nested PCR with vector specific and Sp6 promoter primers
5'atttaggtgacactatag3'
Sp6
T7/Sp6
5'LTR
Primer 5b
5'aatactgacgctctcgcacccatc3'
5'ctgtactgggtctctctgg3'
Primer3b
3'LTR
T7/Sp6
Sp6
5'atttaggtgacactatag3'
6). Subclone into pCR®2.1-TOPO®, sequence with M13 primer(-20) and BLAST search
